# The EU’s Per- and Polyfluoroalkyl Substances (PFAS) Ban: A Case of Policy over Science

**DOI:** 10.3390/toxics11090721

**Published:** 2023-08-22

**Authors:** Francesca Spyrakis, Tommaso A. Dragani

**Affiliations:** 1 Department of Drug Science and Technology, University of Turin, 10125 Turin, Italy; francesca.spyrakis@unito.it; 2 Aspidia srl, 20100 Milan, Italy

**Keywords:** ECHA, PFAS, polyfluoroalkyl, perfluoroalkyl, health, pollution

## Abstract

The proposal by the European Chemicals Agency (ECHA) to ban over 12,000 per- and polyfluoroalkyl substances (PFAS) has sparked a debate about potential consequences for the economy, industry, and the environment. Although some PFAS are known to be harmful, a blanket ban may lead to significant problems in attempting to replace PFAS-based materials for environmental transition, as well as in medical devices and everyday products. Alternative materials may potentially be less safe, as a rush to replace PFAS would reduce the time needed for toxicological analyses. Studies have shown that PFAS exhibit a diverse range of mechanisms of action, biopersistence, and bioaccumulation potential, and should thus not be treated as a single group. This is particularly true for the class of fluoropolymers. A targeted approach that considers the specific risks and benefits of each chemical may be more effective. Moreover, the proposed ban may also have unintended consequences for the environment as PFAS use is also associated with benefits such as reducing greenhouse-gas emissions and improving energy efficiency. Policymakers must carefully weigh up the potential consequences before making a final decision on the ban.

## 1. Introduction

On 7 February 2023, the European Chemicals Agency (ECHA) published a proposal to ban an entire class of chemicals known as per- and polyfluoroalkyl substances (PFAS) [1]. This proposed ban is unprecedented, and, if approved, would affect more than 12,000 PFAS [2]. Based on the revised OECD definitions, ‘PFASs consist of a fully (per) or partly (poly) fluorinated carbon chain connected to different functional groups.’ [3].

However, PFAS are not a chemical class of similar compounds, but the term includes a wide range of compounds with very different physical, chemical, environmental, and biological properties. Although some PFAS are known to be harmful to both the environment and human health, others are not, and the vast majority have yet to be toxicologically characterized. For example, fluoropolymers do not exhibit the toxicological and environmental properties commonly associated with other PFAS of concern [4].

PFAS are widely used in a variety of industries, including textiles, electronics, food packaging, electric car batteries, various household products, cosmetics, pharmaceuticals, pesticides, and medical device manufacturing. A blanket ban on all substances could have serious economic, industrial, and environmental consequences, and, paradoxically, public health implications [5,6]. Moreover, replacing PFAS with alternative substances may be prohibitively expensive and even impossible in some cases.

It is likely that the ban will not be fully implemented for reasons of economic and social sustainability, but the current proposal, if not quickly withdrawn, will lead to a hasty search for substitutes that may perform worse than PFAS, be more expensive, and most likely be less characterized toxicologically.

It is difficult for material chemists to imagine possibly replacing all PFAS, within a few years, with alternative non-fluorinated compounds that have the same chemical and physical properties as the products they are intended to replace.

It should also be noted that the proposed ban on the entire class of PFAS is being proposed in the absence of scientific evidence to prove that the end products made with PFAS are harmful. This is particularly true for fluoropolymers and perfluoropolyether oils, which are used in a wide range of industries and applications, including the automotive, aerospace, chemical, and nuclear industries, and electronics, medical devices, and green-economy initiatives [7,8]. These materials have not been linked to any adverse effects on humans but have rather improved quality of life and well-being.

Finally, a considerable biomonitoring effort in the United States and Australia found that there was no major increase in human exposure to PFAS from 1970 to 2010. Indeed, PFAS exposure has been decreasing in both countries since the early 2000s, and it is reasonable to assume that the downward trend in PFAS exposure will continue there, as well as in European countries [9]. Indeed, a noticeable declining trend in PFAS serum concentrations has been observed among Swedish adolescents and Norwegian men and women [10,11]. This downward trajectory can be attributed to the phased discontinuation of legacy PFAS compounds. The emergence of novel PFAS, presently undergoing production and utilization, presents a potential challenge to conventional biomonitoring endeavors. Consequently, active efforts are currently underway to establish suitable methodologies for the detection of these alternative PFAS compounds within human blood [12]. To date, the investigation of alternative PFAS compounds within human blood has been limited. Few studies have either failed to detect their presence or have identified minimal levels [13,14,15]. Notably, a study conducted in Sweden found an increase in the levels of perfluorobutane sulfonic acid (PFBS) and perfluorohexanesulfonic acid (PFHxS)—two alternative PFAS compounds—in the blood serum of pregnant women from 1996 to 2010 [16]. Interestingly, this increases contrasts with the concurrent decrease in concentrations of PFOS and PFOA over the same period [16].

## 2. PFAS Are Not a Small Group of Chemicals with Similar Properties

**Figure 1:**
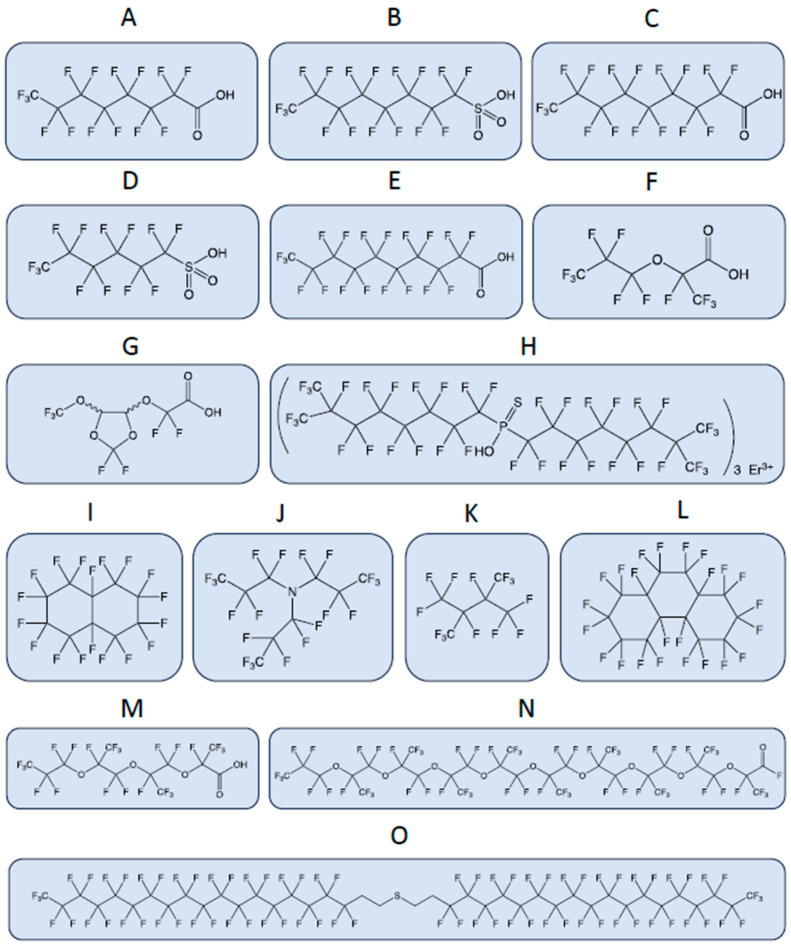
A few examples highlight the huge heterogeneity of chemical structures of PFAS, which poses challenges for their regulation. The physical and chemical properties of PFASs vary widely depending on their chain length, branching, and functional groups. These properties affect their environmental fate and transport, bioaccumulation potential, and toxicity. The bonds between the atoms are represented by lines. CAS, Chemical Abstracts Service Registry Number, is a numeric identifier assigned by the Chemical Abstracts Service (CAS) division of the American Chemical Society (ACS). (**A**) Perfluorooctanoic acid (PFOA), CAS: 335-67-1; (**B**) perfluorooctanesulfonic acid (PFOS), CAS: 1763-23-1; (**C**) perfluorononanoic acid (PFNA), CAS: 375-95-1; (**D**) perfluorohexanesulfonic acid (PFHxS), CAS: 355-46-4; (**E**) perfluorodecanoic acid (PFDA), CAS: 335-76-2; (**F**) 2,3,3,3-Tetrafluoro-2-(heptafluoropropoxy)propanoic acid (GenX or HFPO-DA), CAS: 13252-13-6; (**G**) 2,2-difluoro-2-{[2,2,4,5-tetrafluoro-5-(trifluoromethoxy)-1,3-dioxolan-4-yl]oxy}acetic acid (C6O4), CAS: 682-238-0; (**H**) P,P-Bis(perfluoro-7-methyloctyl) phosphinothioic acid erbium(3+) salt (3:1), CAS: 500776-89-6; (**I**) perfluorodecalin, CAS: 306-94-5; (**J**) perfluorotripropylamine, CAS: 338-83-0; (**K**) perfluoro-2,3-dimethylbutane, CAS: 354-96-1; (**L**) perfluoroperhydrophenanthrene, CAS: 306-91-2; (**M**) perfluoro-(2,5,8-trimethyl-3,6,9-trioxadodecanoic)acid, CAS: 65294-16-8; (**N**) dotriacontafluorononakis(trifluoromethyl)nonaoxatriacontanoyl fluoride, CAS: 65150-88-1; (**O**) bis 18:2 fluorotelomer thioether.

A recent paper on PFAS terminology, developed within the Organization for Economic Cooperation and Development (OECD), highlights the tremendous heterogeneity in the chemical structures of different PFAS (Figure 1) and the need to revise PFAS terminology [17]. The term PFAS, which is commonly used to define the entire chemical class, is too general and likely to cause further confusion. The most studied PFAS that pose toxicological risks to humans and the environment are non-polymeric perfluoroalkyl carboxylic acids (PFCA), e.g., perfluorooctanoic acid, PFOA, and perfluorosulfonic acids (PFSA), e.g., perfluorooctane sulfonic acid and PFOS.

The chemical class of PFAS includes many substances that are uncharacterized to such an extent that their physical and chemical properties, including their solubility in water, are unknown. In fact, for some PFAS, the only available values for physical and chemical properties are estimates derived from mathematical models, such as quantitative structure–activity relationship (QSAR) models [18], which can approximate the chemical and physical properties of compounds based on their chemical structure, rather values from direct measurements [19,20].

At ambient temperature and pressure, long-chain PFAS typically exist in solid form as crystalline or amorphous powders. By contrast, short-chain PFAS, with 4–6 carbon atoms, are generally liquids at room temperature. Available data suggest that both the melting temperature and melting enthalpy of PFAS increase with the length of the fluorinated carbon chain [20,21].

Vapor pressure, which is a measure of the volatility of a compound (the higher the vapor pressure, the more volatile the compound), is particularly relevant in evaluating the potential toxicity of PFAS. Highly volatile compounds have a greater potential for long-range transport because they are easily converted to the gas phase and can travel long distances in the atmosphere, whereas chemicals with low vapor pressures are more likely to remain in the solid and liquid forms and are generally transported through the soil, surface and groundwater, with reduced transport potential [22]. The ambient vapor pressure of PFAS salts is significantly lower than that of the corresponding acidic forms. For example, the vapor pressure of the ammonium salt of PFOA is three orders of magnitude lower than that of its acid form. To accurately estimate the vapor pressure and environmental transport potential of PFAS, it is therefore necessary to determine their exact chemical nature in the environment, as different chemical forms can have very different vapor pressures [23].

The chemical stability of a molecule greatly influences its persistence in the environment [24,25,26]. The polar regions of PFAS, like the acid groups, can be susceptible to numerous chemical transformations. A recent study has shown that the carboxy terminal tail of PFCAs can facilitate a sodium hydroxide-mediated defluorination mechanism. This chemical degradation process occurs in the presence of the solvent dimethylsulfoxide and leads to highly reactive perfluoroalkyl intermediates that undergo further degradation, culminating in the final generation of fluoride ions [27]. It is therefore scientifically inaccurate to consider the tens of thousands of PFAS as a single group of molecules with similar chemical and physical properties, biopersistence, bioaccumulation, and toxicity.

## 3. Different Structures Mean Different Mechanisms of Action

The biochemical action of some PFCA and PFSA, particularly PFOA and PFOS, has been widely characterized. In rodents, these compounds appear to exert toxicological effects by binding to and activating the peroxisome proliferator receptor alpha (PPARα), a transcription factor that regulates lipid metabolism, energy balance and inflammation [28,29,30,31]. However, PPARα activators are unlikely to cause liver tumors in humans due to differences in biological response to PPARα activation in key downstream events [28,32,33].

Results from several studies highlight differences between human and rodent PPARα responses in mediating the toxic effects of PFAS compounds. Collectively, these studies highlight differences in dose–response relationships, target endpoints, and receptor activation thresholds between humans and rodents. Specifically, activation of PPARα target genes by ammonium perflurooctanate (APFO) is prominently observed in the liver of mice with mouse PPARα, whereas such effects are notably absent in mice lacking PPARα or possessing human PPARα [34]. This discrepancy suggests that human PPARα has a relatively low reactivity to APFO, especially at lower doses.

A study on APFO-induced liver injury shows different histopathologic manifestations, supporting the conclusion that APFO activates PPARα differently in mice and humans, potentially leading to different liver injury [29].

The induction of gene transcriptional profiling by PPARα activation is a mechanism that affects rodent but not human liver cells, reinforcing the notion that PPARα-mediated liver toxicity observed in rodents cannot be directly extrapolated to assess human health implications [35].

A study on pregnant mice exposed to PFOA, showed that, on postnatal day 20, wild-type mice exhibited higher relative liver weight and increased hepatic gene expression compared to PPARα-null and PPARα-humanized mice [36]. These findings suggest that prenatal PFOA effects on development depends on mouse and human PPARα differences [36].

The immunomodulatory effects of PFOA in mice with or without PPARα, i.e., the reductions in spleen and thymus weight, along with alterations in cell populations, caused by PFOA treatment, were absent or attenuated in PPARα-null mice. Additionally, the diminished in vitro response of splenocytes from treated mice was not observed in PPARα-null mice. These findings highlight the significant role of PPARα in the immunomodulation induced by PFOA and other peroxisome proliferators [37].

Although PPARα is one receptor that mediates the biochemical and toxicological activities of PFAS, it is not the only one, and not all PFAS activate PPARα. A study by Behr et al. [38] made use of in vitro genetic assays on human cells to determine whether PFOA, PFOS, and six other PFAS are able to activate eight other human nuclear receptors, in addition to PPARα. The results showed that all of the PFAS tested, except PFBS, activated human PPARα. Perfluoro-2-methyl-3-oxahexanoic acid (PMOH) and 3H-perfluoro-3-[(3-methoxypropoxy)propanoic acid] (PMPP) were weak agonists of human PPARγ. The other human nuclear receptors tested (PPARδ, CAR, PXR, FXR, LXRα, RXRα, and RARα) were not activated by any of the tested PFAS. The effects observed in vitro occurred only at PFAS concentrations above 10 μM, i.e., 5001 and 4141 ng/mL for PFOS and PFOA, respectively, which is several orders of magnitude higher than the mean PFAS concentration in the blood of Western populations, for whom mean serum and plasma concentrations were reported to be 7.7 and 1.9 ng/mL for PFOS and PFOA, respectively [39].

Houck et al. (2021) evaluated 142 PFAS in vitro and measured 81 different transcription factor activities. The results showed activity for several nuclear receptors, including three known targets of the characterized PFCA and PFSA: estrogen receptor alpha, PPARα, and PPARγ. In addition, activity was observed against retinoid X receptor beta, the major heterodimeric partner of type II nonsteroidal nuclear receptors, the pregnane X receptor, nuclear receptor-related protein-1, and erythroid nuclear factor 2-related protein-1 [40].

In a study of potential immunosuppressive activity in a panel of 12 primary human cell systems and 148 relevant biomarkers, only 21% of the 147 PFAS analyzed showed bioactivity. The activity profile of PFOS showed little correlation with the reference immunosuppressants, suggesting that in vivo activity may occur via different mechanisms. The activity profile of PFOA shares some common features with that of dexamethasone, but also shows unique characteristics [41].

One of the key factors that contributes to the bioaccumulation of PFCA and PFSA in the human body is their limited excretion by transporters present in the kidneys, which play an active role in reabsorbing PFAS from pre-urine and transporting them back to cells located in the proximal tubule of the renal system [42]. In humans, organic anion transporter 4 (OAT4) and urate transporter 1 (URAT1) play important roles in facilitating PFAS reuptake from the pre-urinary environment [43]. A study by Louise et al. (2023) analyzed the transport of seven PFAS, namely PFHpA, PFOA, PFNA, PFDA, PFBS, PFHxS, and PFOS, in human embryonic kidney (HEK) cells transfected with either URAT1 or OAT4. The results indicated that there was no significant PFAS transport in HEK cells transfected with URAT1. However, all PFAS, except PFBS, were taken up in HEK cells transfected with OAT4 [44]. By contrast, an examination of a new-generation PFAS, C6O4, in two renal cell lines transfected with either URAT1 or OAT4 showed negligible C6O4 uptake under the experimental conditions, whereas both URAT1- and OAT4-transfected cells displayed uptake of the reference PFAS, perfluorohexanoic acid (PFC6). These results highlight the existence of different transport mechanisms for different PFAS, with these mechanisms potentially influencing their elimination and bioaccumulation in the body [45].

In summary, it is important to recognize that the molecular targets and mechanisms underlying PFAS toxicity may vary with the specific PFAS, as well as with the specific tissue and organ examined, and the mammalian species involved. This underscores the need for targeted analyses that will assess the toxicological potential and bioaccumulation potential of different PFAS molecules.

## 4. The Lack of Scientific Basis for the Application of the Toxicity-Equivalent Factors to PFAS

In toxicology, an analysis of the relative potency of structurally related chemicals, when possible, plays an important role, as it allows an estimation of the equivalent doses of target chemicals relative to a reference chemical to be performed. The use of toxic equivalency factors (TEFs) has been proposed as a means of estimating the combined toxicity resulting from exposure to mixtures of chemicals that have significant structural similarities and elicit comparable toxicological responses in different species [46]. For example, TEFs have been used to characterize the toxicity of polychlorinated chemicals because their mechanism of toxicity appears to result from the activation of a specific receptor [47].

TEFs may offer regulators a tool for evaluating the potential toxicity of PFAS mixtures. This is accomplished by determining the overall concentration of PFOA equivalents within a mixture, and comparing it to the specific regulatory limits set for PFOA in various contexts, such as in drinking water and food. Via this method, it becomes possible to estimate population exposure by considering the consumption of contaminated drinking water or food that contains a specific combination of PFAS. This estimation allows for a comparison with the tolerable daily or weekly dose that has been established by regulatory agencies. As a result, an assessment of the health risks associated with oral exposure to a particular mixture can be made. It is important to note, however, that the health risk determined using this approach is constrained to the toxicological parameter used in calculating the TEFs, namely liver weight, and the presumed mechanism of action. In fact, the application of TEFs to PFASs is based on the unproven assumption that their toxic effects are similar and result solely from an identical mechanism of action. Without this fundamental congruence, the proposed TEFs for assessing health risks from mixtures of toxic agents would lack logical consistency. In simpler terms, the formulation of a TEF paradigm that includes agents with different toxic mechanisms and profiles, such as lead, benzo(a)pyrene, and benzene, would not be considered a viable proposal. This is analogous to postulating a scenario in which contaminants belonging to the same broad chemical class, e.g., PFAS, with different mechanisms and toxicities are collectively included in a unified TEF construct.

Recent studies did not modify the limitation of using rodent parameters to establish TEFs that are intended to be used to estimate human health risks related to PFAS mixtures exposure. Indeed, a database of rat liver endpoints for 16 PFAS was established, allowing for relative potency factors (RPFs) calculations and risk assessment of mixture exposure [48]. The same research group derived RPFs for various PFAS at the blood serum level, in male rats. By applying dose–response modeling, these internal exposures are used to derive quantitative internal RPFs based on liver weight effects [49]. A more recent study aimed to establish RPFs for the immune suppressive effects of PFAS using rodent and human data. RPFs were successfully derived for PFAS based on rat lymphoid organ weights and globulin concentration. Seven PFAS were ranked for immunotoxic potency. Epidemiological data indicated inverse associations of the sum of PFOA, PFNA, PFHxS, and PFOS concentration with serum antibody concentration to mumps and rubella, but did not allow reliable RPFs estimation [50].

However, the toxic effects of PFAS may depend on multiple factors, including the bioavailability of each molecule, which is affected by absorption, metabolism, and binding to specific receptors. In addition, binding affinities and receptor interactions can vary among molecules in the same PFAS family.

To overcome the problems associated with estimating TEFs, Colnot and Dekant (2022) proposed a classification strategy for PFAS that divides them into two distinct groups: PFCA and PFSA. The authors concluded that PFAS with short chains or non-linear structures should not be included in either group because of their low toxicological potency and rapid elimination [51].

Evans et al. (2022) conducted an analysis to assess the ability of 16 PFAS compounds to activate PPARα, human and rat PPARγ, and other receptors. Interestingly, in vitro measurements of PPARα and PPARγ activity in human and rat models did not correlate with the oral doses or serum concentrations of PFAS that were associated with increased liver weights in male rats, as observed in National Toxicology Program 28-day toxicity studies [52].

Another study analyzed the toxicokinetics of five PFAS compounds in various mammalian species, focusing on tissue distribution, half-life, and transfer to developing offspring via placental transfer and lactation. The study reviewed a comprehensive set of 70 studies in the literature that provided quantitative toxicokinetic information for at least one of the five PFAS compounds in different mammalian species. While extensive data are available on the absorption, distribution, metabolism, and excretion of PFOA and PFOS in both humans and animals, limited information is available for PFHxS, PFBS, and PFBA. Despite these limitations, the results of the study showed there are significant interspecies differences in some of the toxicokinetic parameters of different PFAS, raising questions as to whether the substances can be regulated as a single group. In addition, the study highlighted the significant problems in extrapolating health effects from laboratory animals to humans in the context of PFAS exposure [42].

Overall, given the existing differences in toxicokinetics and mechanisms of action, and the lack of comprehensive data, the development of a TEF approach for perfluoroalkyl chemicals remains a distant goal rather than a scientifically established outcome [53,54,55].

## 5. Bioaccumulation of PFAS: The Role of Chain Length

Bioaccumulation or biopersistence refers to the accumulation of a substance in an organism over time. Obviously, the bioavailability of a substance is a prerequisite for bioaccumulation. PFCAs and PFSAs, but not all PFAS, exhibit high stability and their lipophilicity depends on the length of the alkylic chain, resulting in their accumulation in several tissues [56]. Moreover, they can bind to human serum albumin and other transporters in the blood [57]. These properties make PFCA and PFSA potentially able to bioaccumulate in humans and animals.

The bioaccumulation of PFCAs and PFSAs is, indeed, influenced by their chemical structure. Compounds with longer carbon chains, such as PFOA and PFOS, are the most persistent in the environment and can accumulate in living organisms [26,58,59]. By contrast, short-chain PFAS are less likely to bioaccumulate [59,60,61,62]. In fact, although short-chain PFAS have been detected in aquatic systems, their concentrations are generally lower than those of long-chain PFAS [63]. In particular, long-chain PFAS are more likely to accumulate in the brain than short-chain PFAS due to their ability to cross brain barriers [64].

A study analyzing PFAS profiles in drinking water and biological samples from airport workers exposed to contaminated groundwater found that ‘historical’ PFAS accounted for 50% of the total PFAS in drinking water and 90% in serum. Branched PFOS isomers had shorter half-lives than linear PFOS isomers, with half-lives generally decreasing with decreasing chain length [65].

Fluoropolymers, on the other hand, do not pose a bioaccumulation risk because their high molecular weight prevents their absorption by the body, and thus their bioavailability [66].

## 6. Fluoropolymers Are a Separate Class from Smaller PFAS Molecules

Although they fall into the PFAS category, fluoropolymers are a distinct class of chemical compounds characterized by much larger molecular sizes (typical molecular weights > 100,000 Da) and more complex structures than the smaller PFAS molecules. Fluoropolymers consist of long carbon chains with multiple repeating units and fluorine atoms, occasionally accompanied by branching or cross-linking between polymer chains. Compared to small PFAS molecules, the larger size and often complex structures of fluoropolymers likely limit their uptake by living organisms, thereby reducing their likelihood of bioaccumulation. In addition, the large size of fluoropolymers results in their lower solubility in water, further limiting their mobility and potential for dispersion in the environment [66,67]. In fact, fluoropolymers can be classified as low-risk polymers (PLCs), as they meet all the requirements for this classification [68,69].

Overall, size, structure, and water solubility play a key role in determining the biological fate and potential damage of fluorinated substances [66].

### Size Limits for Small-Molecule Biological Activity

Size plays, along with charge and structure, a critical role in determining the penetration of molecules across cell membranes. In the development of new drugs, 500 Da is often quoted as the maximum molecular weight parameter. However, it has been observed that molecules with higher molecular weights are also capable of being absorbed, and the limits of oral bioavailability appear to extend to about MW ≤ 1000 Da [70,71,72].

Thus, data accumulated from extensive investigations of various pharmacological and non-pharmacological substances indicate that molecules with molecular weights above 1000 Da have very little, if any, ability to diffuse across cell membranes and, as a result, are not bioavailable when taken orally.

Therefore, substances with molecular weights greater than 1000 Da, such as fluoropolymers, which generally have molecular weights much greater than 1000 Da, have negligible bioavailability, resulting in limited potential toxicity and bioaccumulation.

## 7. Bioremediation of PFAS: Challenges and Opportunities

Recent advances in PFAS degradation via thermal and non-thermal methods have been recently reviewed. Along with physicochemical techniques [27], bioremediation appears to be a successful solution for PFAS removal from the environment [73,74].

Bioremediation is a process that utilizes the metabolic capabilities of microorganisms to degrade and detoxify contaminants. The microbial degradation of PFAS is emerging as a promising approach for the remediation of contaminated waters and sites. For example, *Acidimicrobium* sp. strain A6 is capable of defluorinating PFOA and PFOS through a reaction in which iron is reduced and ammonium or hydrogen are used as electron donors; this reaction leads to the formation of shorter-chain perfluorinated products and acetate [75]. Another study investigated the role of carbon–carbon double bonds in the biodegradation of unsaturated PFAS, showing that α,β unsaturation is critical for anaerobic reductive defluorination and highlighting the enhanced degradability of unsaturated fluorinated carboxylic acids with α/β-trifluoromethyl branches [76]. Several microbial enzymes, including esterases, hydrolases, oxidases, reductases, and dehalogenases, play key roles in PFAS biodegradation, and advances in enzyme engineering and biocatalysis offer the potential for the development of efficient and sustainable PFAS bioremediation strategies [77,78].

However, the diversity of PFAS structures poses a challenge for bioremediation. Long-chain PFCAs and PFSAs may be more resistant to biodegradation than their short-chain counterparts. Despite these challenges, bioremediation offers several advantages over other remediation methods, such as chemical treatment and incineration. Bioremediation has very low costs and is environmentally friendly because it does not require expensive equipment and does not produce harmful byproducts [79].

More research is needed to determine the feasibility of bioremediation as an effective strategy for PFAS remediation and to optimize the degradation of PFAS with different chemical structures. In our opinion, should PFAS bioremediation techniques demonstrate their effectiveness, the depiction in media and the emphasis in regulatory proposals that currently categorize PFAS contaminants as ‘forever chemicals’ to underscore their environmental risk may need to be reevaluated.

## 8. Unintended Consequences of the Proposed PFAS Ban

The European Union’s proposed ban on PFAS may have unintended consequences and may not necessarily lead to safer alternatives. To mitigate the ban on the entire chemical class of PFAS, the concept of ‘essential use’ has been proposed to identify which PFAS can be phased out. However, there are several challenges inherent in this approach, with these including a lack of comprehensive data on potential substitutes, the complexity of supply chains, product formulation, and product disposal [80].

In addition, the availability of viable alternatives is limited. PFAS have unique properties, such as exceptional water repellency and oleophobicity, that make them advantageous in a variety of applications. Identifying substitutes that can match the performance of PFAS has proven difficult. For example, in the field of medical devices, fluoropolymer-based structures offer a superior and safer alternative to devices made from other types of polymers; when polypropylene and polymethylpentene are used to make artificial lung membranes for blood oxygenation during open-heart surgery or acute lung failure, they often exhibit inadequate biocompatibility, resulting in unwanted blood clotting and long-term hemolysis. By contrast, a novel fluoropolymer-based membrane has demonstrated optimal performance with no detectable hemolysis and complete biocompatibility. This highlights the significant utility of fluoropolymers in medical-device manufacturing [81].

The substitution of PFAS with alternative chemicals is not necessarily safer. Chemical substitution is common in the industry, but the safety of substitute chemicals is often only partially known in the early days of market introduction, and potential PFAS substitutes may pose new risks to humans and the environment.

The proposed ban may also have unintended environmental consequences if it includes fluoropolymers and their precursors, as several fluoropolymers contribute to green-transition technologies (e.g., lithium batteries, various materials for electric vehicles, perfluoro ionomers for fuel cells and electrolyzers for hydrogen production), meaning that the stated benefits could be negated if the PFAS ban results in the use of more toxic or less effective alternatives.

## 9. Conclusions

In summary, the proposed European ban on more than 12,000 PFAS raises several concerns and potential unintended consequences. While some PFAS are known to be harmful to the environment and human health, a blanket ban on all of them may not be the most effective and sustainable solution. The diverse range of PFAS chemical structures, mechanisms of action, biopersistence, and bioaccumulation potential makes their treatment as a single group scientifically unsound. Fluoropolymers differ significantly from other PFAS in terms of their chemical properties, bioaccumulation potential, and toxicity. They pose little toxicological risk as they are much less bioavailable and bioaccumulative, and there is no strong scientific basis for their inclusion in the ban.

In addition, any decision must also consider a lack of viable and safe alternatives, and the risk of reduced effectiveness of health care for European citizens due to a lack of essential medical device materials. The possibility that the ban may also affect or significantly slow the green transition must also be considered.

The ban could force the use of alternative chemicals that are not well-toxicologically characterized and may be more toxic than PFAS.

PFAS may cease to be considered ‘forever chemicals’ if scientific research on bioremediation develops and allows for the remediation of PFAS at a very low cost.

Ultimately, a more balanced and focused approach that centers on the regulation and management of high-risk PFAS, while supporting the research and development of safer remediation alternatives and technologies, should be considered. This would result in more effective protection of human health and the environment, while minimizing any potentially harmful impact on the health and quality of life of European citizens.

## Data Availability

Not applicable.

## References

[1] ECHA Annex XV Restriction Report: Proposal for a Restriction. https://echa.europa.eu/documents/10162/f605d4b5-7c17-7414-8823-b49b9fd43aea.

[2] EPA PFAS Master List of PFAS Substances. https://comptox.epa.gov/dashboard/chemical-lists/pfasmaster.

[3] OECD What Are PFASS and What Are They Used for?. https://www.oecd.org/chemicalsafety/portal-perfluorinated-chemicals/aboutpfass/.

[4] Ameduri B. (2023). Fluoropolymers: A Special Class of per- and Polyfluoroalkyl Substances (PFASs) Essential for Our Daily Life. J. Fluor. Chem..

[5] Chetty R., Stepner M., Abraham S., Lin S., Scuderi B., Turner N., Bergeron A., Cutler D. (2016). The Association Between Income and Life Expectancy in the United States, 2001–2014. JAMA.

[6] Saito M., Kondo N., Oshio T., Tabuchi T., Kondo K. (2019). Relative Deprivation, Poverty, and Mortality in Japanese Older Adults: A Six-Year Follow-Up of the JAGES Cohort Survey. Int. J. Environ. Res. Public Health.

[7] Lv J., Cheng Y. (2021). Fluoropolymers in Biomedical Applications: State-of-the-Art and Future Perspectives. Chem. Soc. Rev..

[8] Wang M., Tsuda M., Deguchi S., Higuchi Y., So K., Torisawa Y.-S., Takayama K., Yamashita F. (2022). Application of Perfluoropolyether Elastomers in Microfluidic Drug Metabolism Assays. Int. J. Pharm..

[9] Gomis M.I., Vestergren R., MacLeod M., Mueller J.F., Cousins I.T. (2017). Historical Human Exposure to Perfluoroalkyl Acids in the United States and Australia Reconstructed from Biomonitoring Data Using Population-Based Pharmacokinetic Modelling. Environ. Int..

[10] Norén E., Lindh C., Glynn A., Rylander L., Pineda D., Nielsen C. (2021). Temporal Trends, 2000-2017, of Perfluoroalkyl Acid (PFAA) Concentrations in Serum of Swedish Adolescents. Environ. Int..

[11] Berg V., Sandanger T.M., Hanssen L., Rylander C., Nøst T.H. (2021). Time Trends of Perfluoroalkyl Substances in Blood in 30-Year Old Norwegian Men and Women in the Period 1986–2007. Environ. Sci. Pollut. Res. Int..

[12] Frigerio G., Cafagna S., Polledri E., Mercadante R., Fustinoni S. (2022). Development and Validation of an LC-MS/MS Method for the Quantitation of 30 Legacy and Emerging per- and Polyfluoroalkyl Substances (PFASs) in Human Plasma, Including HFPO-DA, DONA, and CC6O4. Anal. Bioanal. Chem..

[13] Feng X., Chen X., Yang Y., Yang L., Zhu Y., Shan G., Zhu L., Zhang S. (2021). External and Internal Human Exposure to PFOA and HFPOs around a Mega Fluorochemical Industrial Park, China: Differences and Implications. Environ. Int..

[14] Petriello M.C., Mottaleb M.A., Serio T.C., Balyan B., Cave M.C., Pavuk M., Birnbaum L.S., Morris A.J. (2022). Serum Concentrations of Legacy and Emerging Per- and Polyfluoroalkyl Substances in the Anniston Community Health Surveys (ACHS I and ACHS II). Environ. Int..

[15] Kotlarz N., McCord J., Collier D., Lea C.S., Strynar M., Lindstrom A.B., Wilkie A.A., Islam J.Y., Matney K., Tarte P. (2020). Measurement of Novel, Drinking Water-Associated PFAS in Blood from Adults and Children in Wilmington, North Carolina. Environ. Health Perspect..

[16] Glynn A., Berger U., Bignert A., Ullah S., Aune M., Lignell S., Darnerud P.O. (2012). Perfluorinated Alkyl Acids in Blood Serum from Primiparous Women in Sweden: Serial Sampling during Pregnancy and Nursing, and Temporal Trends 1996-2010. Environ. Sci. Technol..

[17] Wang Z., Buser A.M., Cousins I.T., Demattio S., Drost W., Johansson O., Ohno K., Patlewicz G., Richard A.M., Walker G.W. (2021). A New OECD Definition for Per- and Polyfluoroalkyl Substances. Environ. Sci. Technol..

[18] Polishchuk P. (2017). Interpretation of Quantitative Structure-Activity Relationship Models: Past, Present, and Future. J. Chem. Inf. Model..

[19] Brusseau M.L., Van Glubt S. (2021). The Influence of Molecular Structure on PFAS Adsorption at Air-Water Interfaces in Electrolyte Solutions. Chemosphere.

[20] Lampic A., Parnis J.M. (2020). Property Estimation of Per- and Polyfluoroalkyl Substances: A Comparative Assessment of Estimation Methods. Environ. Toxicol. Chem..

[21] Zhang M., Yamada K., Bourguet S., Guelfo J., Suuberg E.M. (2020). Vapor Pressure of Nine Perfluoroalkyl Substances (PFASs) Determined Using the Knudsen Effusion Method. J. Chem. Eng. Data.

[22] Schindler B.J., Buchanan J.H., Mahle J.J., Peterson G.W., Glover T.G. (2013). Ambient Temperature Vapor Pressure and Adsorption Capacity for (Perfluorooctyl) Ethylene, 3-(Perfluorobutyl)Propanol, Perfluorohexanoic Acid, Ethyl Perfluorooctanoate, and Perfluoro-3,6-Dioxaheptanoic Acid. J. Chem. Eng. Data.

[23] Barton C.A., Botelho M.A., Kaiser M.A. (2009). Solid Vapor Pressure and Enthalpy of Sublimation for Ammonium Perfluorooctanoate. J. Chem. Eng. Data.

[24] Kirsch P. (2013). Modern Fluoroorganic Chemistry: Synthesis, Reactivity, Applications.

[25] Xing Y., Li Q., Chen X., Huang B., Ji L., Zhang Q., Fu X., Li T., Wang J. (2023). PFASs in Soil: How They Threaten Human Health through Multiple Pathways and Whether They Are Receiving Adequate Concern. J. Agric. Food Chem..

[26] Ahrens L., Bundschuh M. (2014). Fate and Effects of Poly- and Perfluoroalkyl Substances in the Aquatic Environment: A Review. Environ. Toxicol. Chem..

[27] Trang B., Li Y., Xue X.-S., Ateia M., Houk K.N., Dichtel W.R. (2022). Low-Temperature Mineralization of Perfluorocarboxylic Acids. Science.

[28] Elcombe C.R., Elcombe B.M., Foster J.R., Farrar D.G., Jung R., Chang S.-C., Kennedy G.L., Butenhoff J.L. (2010). Hepatocellular Hypertrophy and Cell Proliferation in Sprague-Dawley Rats Following Dietary Exposure to Ammonium Perfluorooctanoate Occurs through Increased Activation of the Xenosensor Nuclear Receptors PPARα and CAR/PXR. Arch. Toxicol..

[29] Nakagawa T., Ramdhan D.H., Tanaka N., Naito H., Tamada H., Ito Y., Li Y., Hayashi Y., Yamagishi N., Yanagiba Y. (2012). Modulation of Ammonium Perfluorooctanoate-Induced Hepatic Damage by Genetically Different PPARα in Mice. Arch. Toxicol..

[30] Tyagi S., Gupta P., Saini A.S., Kaushal C., Sharma S. (2011). The Peroxisome Proliferator-Activated Receptor: A Family of Nuclear Receptors Role in Various Diseases. J. Adv. Pharm. Technol. Res..

[31] Gross B., Pawlak M., Lefebvre P., Staels B. (2017). PPARs in Obesity-Induced T2DM, Dyslipidaemia and NAFLD. Nat. Rev. Endocrinol..

[32] Corton J.C., Peters J.M., Klaunig J.E. (2018). The PPARα-Dependent Rodent Liver Tumor Response Is Not Relevant to Humans: Addressing Misconceptions. Arch. Toxicol..

[33] IARC (1995). Peroxisome Proliferation and Its Role in Carcinogenesis.

[34] Nakamura T., Ito Y., Yanagiba Y., Ramdhan D.H., Kono Y., Naito H., Hayashi Y., Li Y., Aoyama T., Gonzalez F.J. (2009). Microgram-Order Ammonium Perfluorooctanoate May Activate Mouse Peroxisome Proliferator-Activated Receptor Alpha, but Not Human PPARalpha. Toxicology.

[35] Bjork J.A., Wallace K.B. (2009). Structure-Activity Relationships and Human Relevance for Perfluoroalkyl Acid-Induced Transcriptional Activation of Peroxisome Proliferation in Liver Cell Cultures. Toxicol. Sci..

[36] Albrecht P.P., Torsell N.E., Krishnan P., Ehresman D.J., Frame S.R., Chang S.-C., Butenhoff J.L., Kennedy G.L., Gonzalez F.J., Peters J.M. (2013). A Species Difference in the Peroxisome Proliferator-Activated Receptor α-Dependent Response to the Developmental Effects of Perfluorooctanoic Acid. Toxicol. Sci..

[37] Yang Q., Xie Y., Alexson S.E.H., Nelson B.D., DePierre J.W. (2002). Involvement of the Peroxisome Proliferator-Activated Receptor Alpha in the Immunomodulation Caused by Peroxisome Proliferators in Mice. Biochem. Pharmacol..

[38] Behr A.-C., Plinsch C., Braeuning A., Buhrke T. (2020). Activation of Human Nuclear Receptors by Perfluoroalkylated Substances (PFAS). Toxicol. In Vitro.

[39] Schrenk D., Bignami M., Bodin L., Chipman J.K., Del Mazo J., Grasl-Kraupp B., Hogstrand C., Hoogenboom L.R., Leblanc J.-C., Nebbia C.S. (2020). Risk to Human Health Related to the Presence of Perfluoroalkyl Substances in Food. EFSA J. Eur. Food Saf. Auth..

[40] Houck K.A., Patlewicz G., Richard A.M., Williams A.J., Shobair M.A., Smeltz M., Clifton M.S., Wetmore B., Medvedev A., Makarov S. (2021). Bioactivity Profiling of Per- and Polyfluoroalkyl Substances (PFAS) Identifies Potential Toxicity Pathways Related to Molecular Structure. Toxicology.

[41] Houck K.A., Friedman K.P., Feshuk M., Patlewicz G., Smeltz M., Clifton M.S., Wetmore B.A., Velichko S., Berenyi A., Berg E.L. (2023). Evaluation of 147 Perfluoroalkyl Substances for Immunotoxic and Other (Patho)Physiological Activities through Phenotypic Screening of Human Primary Cells. ALTEX.

[42] Pizzurro D.M., Seeley M., Kerper L.E., Beck B.D. (2019). Interspecies Differences in Perfluoroalkyl Substances (PFAS) Toxicokinetics and Application to Health-Based Criteria. Regul. Toxicol. Pharmacol..

[43] Yang C.-H., Glover K.P., Han X. (2010). Characterization of Cellular Uptake of Perfluorooctanoate via Organic Anion-Transporting Polypeptide 1A2, Organic Anion Transporter 4, and Urate Transporter 1 for Their Potential Roles in Mediating Human Renal Reabsorption of Perfluorocarboxylates. Toxicol. Sci..

[44] Louisse J., Dellafiora L., van den Heuvel J.J.M.W., Rijkers D., Leenders L., Dorne J.-L.C.M., Punt A., Russel F.G.M., Koenderink J.B. (2023). Perfluoroalkyl Substances (PFASs) Are Substrates of the Renal Human Organic Anion Transporter 4 (OAT4). Arch. Toxicol..

[45] Bruno S., Bersani M., Astore S., Chiabotto G., Barge A., Binello A., Spyrakis F. (2022). Lack of Interaction of the Fluorosurfactant C6O4 with Human Renal Transporters: In Vitro/in Silico Analysis. Toxicology.

[46] Van den Berg M., Birnbaum L.S., Denison M., De Vito M., Farland W., Feeley M., Fiedler H., Hakansson H., Hanberg A., Haws L. (2006). The 2005 World Health Organization Reevaluation of Human and Mammalian Toxic Equivalency Factors for Dioxins and Dioxin-like Compounds. Toxicol. Sci..

[47] Bradshaw T.D., Bell D.R. (2009). Relevance of the Aryl Hydrocarbon Receptor (AhR) for Clinical Toxicology. Clin. Toxicol..

[48] Bil W., Zeilmaker M., Fragki S., Lijzen J., Verbruggen E., Bokkers B. (2021). Risk Assessment of Per- and Polyfluoroalkyl Substance Mixtures: A Relative Potency Factor Approach. Environ. Toxicol. Chem..

[49] Bil W., Zeilmaker M.J., Bokkers B.G.H. (2022). Internal Relative Potency Factors for the Risk Assessment of Mixtures of Per- and Polyfluoroalkyl Substances (PFAS) in Human Biomonitoring. Environ. Health Perspect..

[50] Bil W., Ehrlich V., Chen G., Vandebriel R., Zeilmaker M., Luijten M., Uhl M., Marx-Stoelting P., Halldorsson T.I., Bokkers B. (2023). Internal Relative Potency Factors Based on Immunotoxicity for the Risk Assessment of Mixtures of Per- and Polyfluoroalkyl Substances (PFAS) in Human Biomonitoring. Environ. Int..

[51] Colnot T., Dekant W. (2022). Commentary: Cumulative Risk Assessment of Perfluoroalkyl Carboxylic Acids and Perfluoralkyl Sulfonic Acids: What Is the Scientific Support for Deriving Tolerable Exposures by Assembling 27 PFAS into 1 Common Assessment Group?. Arch. Toxicol..

[52] Evans N., Conley J.M., Cardon M., Hartig P., Medlock-Kakaley E., Gray L.E.J. (2022). In Vitro Activity of a Panel of Per- and Polyfluoroalkyl Substances (PFAS), Fatty Acids, and Pharmaceuticals in Peroxisome Proliferator-Activated Receptor (PPAR) Alpha, PPAR Gamma, and Estrogen Receptor Assays. Toxicol. Appl. Pharmacol..

[53] Peters J.M., Gonzalez F.J. (2011). Why Toxic Equivalency Factors Are Not Suitable for Perfluoroalkyl Chemicals. Chem. Res. Toxicol..

[54] Ojo A.F., Peng C., Ng J.C. (2021). Assessing the Human Health Risks of Per- and Polyfluoroalkyl Substances: A Need for Greater Focus on Their Interactions as Mixtures. J. Hazard. Mater..

[55] Goodrum P.E., Anderson J.K., Luz A.L., Ansell G.K. (2021). Application of a Framework for Grouping and Mixtures Toxicity Assessment of PFAS: A Closer Examination of Dose-Additivity Approaches. Toxicol. Sci..

[56] Greaves A.K., Letcher R.J., Sonne C., Dietz R., Born E.W. (2012). Tissue-Specific Concentrations and Patterns of Perfluoroalkyl Carboxylates and Sulfonates in East Greenland Polar Bears. Environ. Sci. Technol..

[57] Crisalli A.M., Cai A., Cho B.P. (2023). Probing the Interactions of Perfluorocarboxylic Acids of Various Chain Lengths with Human Serum Albumin: Calorimetric and Spectroscopic Investigations. Chem. Res. Toxicol..

[58] Hsu J.-Y., Hsu J.-F., Ho H.-H., Chiang C.-F., Liao P.-C. (2013). Background Levels of Persistent Organic Pollutants in Humans from Taiwan: Perfluorooctane Sulfonate and Perfluorooctanoic Acid. Chemosphere.

[59] Xu B., Qiu W., Du J., Wan Z., Zhou J.L., Chen H., Liu R., Magnuson J.T., Zheng C. (2022). Translocation, Bioaccumulation, and Distribution of Perfluoroalkyl and Polyfluoroalkyl Substances (PFASs) in Plants. iScience.

[60] Liang X., Yang X., Jiao W., Zhou J., Zhu L. (2022). Simulation Modelling the Structure Related Bioaccumulation and Biomagnification of Per- and Polyfluoroalkyl Substances in Aquatic Food Web. Sci. Total Environ..

[61] Cheng W., Doering J.A., LaLone C., Ng C. (2021). Integrative Computational Approaches to Inform Relative Bioaccumulation Potential of Per- and Polyfluoroalkyl Substances Across Species. Toxicol. Sci..

[62] Lewis A.J., Yun X., Spooner D.E., Kurz M.J., McKenzie E.R., Sales C.M. (2022). Exposure Pathways and Bioaccumulation of Per- and Polyfluoroalkyl Substances in Freshwater Aquatic Ecosystems: Key Considerations. Sci. Total Environ..

[63] Li F., Duan J., Tian S., Ji H., Zhu Y., Wei Z., Zhao D. (2020). Short-Chain per- and Polyfluoroalkyl Substances in Aquatic Systems: Occurrence, Impacts and Treatment. Chem. Eng. J..

[64] Cao Y., Ng C. (2021). Absorption, Distribution, and Toxicity of per- and Polyfluoroalkyl Substances (PFAS) in the Brain: A Review. Environ. Sci. Process. Impacts.

[65] Xu Y., Fletcher T., Pineda D., Lindh C.H., Nilsson C., Glynn A., Vogs C., Norström K., Lilja K., Jakobsson K. (2020). Serum Half-Lives for Short- and Long-Chain Perfluoroalkyl Acids after Ceasing Exposure from Drinking Water Contaminated by Firefighting Foam. Environ. Health Perspect..

[66] Korzeniowski S.H., Buck R.C., Newkold R.M., El Kassmi A., Laganis E., Matsuoka Y., Dinelli B., Beauchet S., Adamsky F., Weilandt K. (2023). A Critical Review of the Application of Polymer of Low Concern Regulatory Criteria to Fluoropolymers II: Fluoroplastics and Fluoroelastomers. Integr. Environ. Assess. Manag..

[67] Henry B.J., Carlin J.P., Hammerschmidt J.A., Buck R.C., Buxton L.W., Fiedler H., Seed J., Hernandez O. (2018). A Critical Review of the Application of Polymer of Low Concern and Regulatory Criteria to Fluoropolymers. Integr. Environ. Assess. Manag..

[68] OECD. Chemicals Committee (2009). Data Analysis of the Identification of Correlations between Polymer Characteristics and Potential for Health or Ecotoxicological Concern.

[69] AICIS Polymer of Low Concern. https://www.industrialchemicals.gov.au/glossary/polymer-low-concern.

[70] Veber D.F., Johnson S.R., Cheng H.-Y., Smith B.R., Ward K.W., Kopple K.D. (2002). Molecular Properties That Influence the Oral Bioavailability of Drug Candidates. J. Med. Chem..

[71] Doak B.C., Over B., Giordanetto F., Kihlberg J. (2014). Oral Druggable Space beyond the Rule of 5: Insights from Drugs and Clinical Candidates. Chem. Biol..

[72] Matsson P., Kihlberg J. (2017). How Big Is Too Big for Cell Permeability?. J. Med. Chem..

[73] Verma S., Lee T., Sahle-Demessie E., Ateia M., Nadagouda M.N. (2022). Recent Advances on PFAS Degradation via Thermal and Nonthermal Methods. Chem. Eng. J. Adv..

[74] Zhang Z., Sarkar D., Biswas J.K., Datta R. (2022). Biodegradation of Per- and Polyfluoroalkyl Substances (PFAS): A Review. Bioresour. Technol..

[75] Huang S., Jaffé P.R. (2019). Defluorination of Perfluorooctanoic Acid (PFOA) and Perfluorooctane Sulfonate (PFOS) by Acidimicrobium Sp. Strain A6. Environ. Sci. Technol..

[76] Yu Y., Che S., Ren C., Jin B., Tian Z., Liu J., Men Y. (2022). Microbial Defluorination of Unsaturated Per- and Polyfluorinated Carboxylic Acids under Anaerobic and Aerobic Conditions: A Structure Specificity Study. Environ. Sci. Technol..

[77] Berhanu A., Mutanda I., Taolin J., Qaria M.A., Yang B., Zhu D. (2023). A Review of Microbial Degradation of Per- and Polyfluoroalkyl Substances (PFAS): Biotransformation Routes and Enzymes. Sci. Total Environ..

[78] Tang K.H.D., Kristanti R.A. (2022). Bioremediation of Perfluorochemicals: Current State and the Way Forward. Bioprocess Biosyst. Eng..

[79] Mishra B., Varjani S., Agrawal D.C., Mandal S.K., Ngo H.H., Taherzadeh M.J., Chang J.-S., You S., Guo W. (2020). Engineering Biocatalytic Material for the Remediation of Pollutants: A Comprehensive Review. Environ. Technol. Innov..

[80] Cousins I.T., De Witt J.C., Glüge J., Goldenman G., Herzke D., Lohmann R., Miller M., Ng C.A., Patton S., Scheringer M. (2021). Finding Essentiality Feasible: Common Questions and Misinterpretations Concerning the “Essential-Use” Concept. Environ. Sci. Process. Impacts.

[81] Park A., Song Y., Yi E., Duy Nguyen B.T., Han D., Sohn E., Park Y., Jung J., Lee Y.M., Cho Y.H. (2020). Blood Oxygenation Using Fluoropolymer-Based Artificial Lung Membranes. ACS Biomater. Sci. Eng..

